# Prevalence of coronary calcification on preoperative computed tomography and its management in thoracic surgery

**DOI:** 10.1007/s00595-022-02532-5

**Published:** 2022-06-13

**Authors:** Ryusuke Machino, Koichiro Shimoyama, Koji Oku, Kazumi Yamasaki, Tsutomu Tagawa

**Affiliations:** 1 Department of Thoracic Surgery, National Hospital Organization Nagasaki Medical Center, 2-1001-1 Kubaru, Ōmura, Nagasaki 856-8562 Japan; 2 Department of Cardiology, National Hospital Organization Nagasaki Medical Center, Ōmura, Japan; 3 Clinical Research Center, National Hospital Organization Nagasaki Medical Center, Ōmura, Japan

**Keywords:** Pulmonary resection, Plain CT, Coronary artery calcification

## Abstract

**Purpose:**

We investigated the preoperative assessment of coronary artery calcification using computed tomography for appropriate intraoperative management to reduce the risk of perioperative cardiac complications during pulmonary resection.

**Methods:**

Patients (*n* = 665) who underwent anatomical lung resection were examined. The extent of preoperative asymptomatic coronary artery stenosis or cardiac complications in patients with coronary artery calcification was assessed. In addition, the risk factors for perioperative cardiac complications were determined.

**Results:**

Coronary artery calcification was detected in 233 (35.0%) asymptomatic patients. Nineteen (8.2%) patients with coronary artery calcification had coronary artery stenosis ≥ 75%. Percutaneous coronary intervention was performed preoperatively (*n* = 3) and postoperatively (*n* = 10), and preoperative drug intervention was performed in 10 cases. One case of severe postoperative cardiac complications and 20 cases of mild postoperative cardiac complications, including those without coronary artery calcification, occurred. Patients with calcified coronary arteries were at risk of cardiovascular complications in the perioperative period. However, patients with coronary artery calcification who underwent preoperative cardiology intervention had no significant perioperative cardiovascular complications.

**Conclusions:**

Coronary artery calcification detected on preoperative computed tomography is a risk factor for perioperative cardiovascular complications. Early intervention may reduce the risk of such complications.

## Introduction

Recently, the number of patients with asymptomatic ischemic heart disease has increased with the increasing number of older adults eligible for surgery. A 2009 study by the Japanese Association of Thoracic Surgery showed that ischemic cardiac diseases occurred in 1350 of 31,301 patients who underwent thoracic surgery in Japan. Furthermore, ischemic heart diseases were identified as the second-most common risk factor for preoperative comorbidity in patients who underwent resection of primary lung cancer caused by smoking [1]. In addition, a 2015 study by the Japanese Association of Thoracic Surgery stated that cardiovascular diseases were the cause of 21 (6.6%) of 318 hospital deaths among 40,302 patients who underwent pulmonary resection for lung cancer [2].

The results of the ISCHEMIA clinical trial showed that there was no significant difference in the incidence of adverse events, such as ischemic cardiovascular events or death from any cause, over a median of 3.2 years between patients that received invasive and conservative treatment for stable coronary disease and moderate or severe ischemia [3]. Therefore, with appropriate intervention, stable ischemia may be managed non-invasively. In contrast, pulmonary resection may affect tt hemodynamics and induce cardiac disease.

Given the above, we hypothesized that perioperative mortality and morbidity could be reduced if we could detect and start treating asymptomatic ischemic heart disease in the preoperative period. Tt evaluation of coronary artery stenosis using coronary artery computed tomography (CT) or coronary angiography (CAG) is just an indication and cannot be applied in all patients undergoing surgery. Interventionalists recently attempted to evaluate the risk of asymptomatic coronary disease by evaluating coronary artery calcification (CAC) using chest CT [4]. The CAC score is calculated as the area and weight of the CAC multiplied by the score on CT images with a 3-mm slice thickness. This score indicates the level of accumulating arteriosclerosis non-invasively, which was reported to increase with the incidence of heart accidents in asymptomatic patients [4]. In a pooled analysis, an increasing CAC score demonstrated a higher perioperative risk, with a CAC score ≥ 1000 conferring the highest risk in non-cardiac surgery cases [5].

As chest CT is essential prior to thoracic surgery, we investigated the clinical significance of preoperatively assessing CAC using plain chest CT and considered appropriate perioperative and intraoperative risk-aversive approaches to manage patients with indications for thoracic surgery.

## Methods

### Study design

This study was approved by the Research Review Board of the National Hospital Organization Nagasaki Medical Center in accordance with the Declaration of Helsinki (approval no.: 2020135, approved on January 4, 2021). Written informed consent for the publication of this report and use of the accompanying images was obtained from all the patients.

**Fig. 1:**
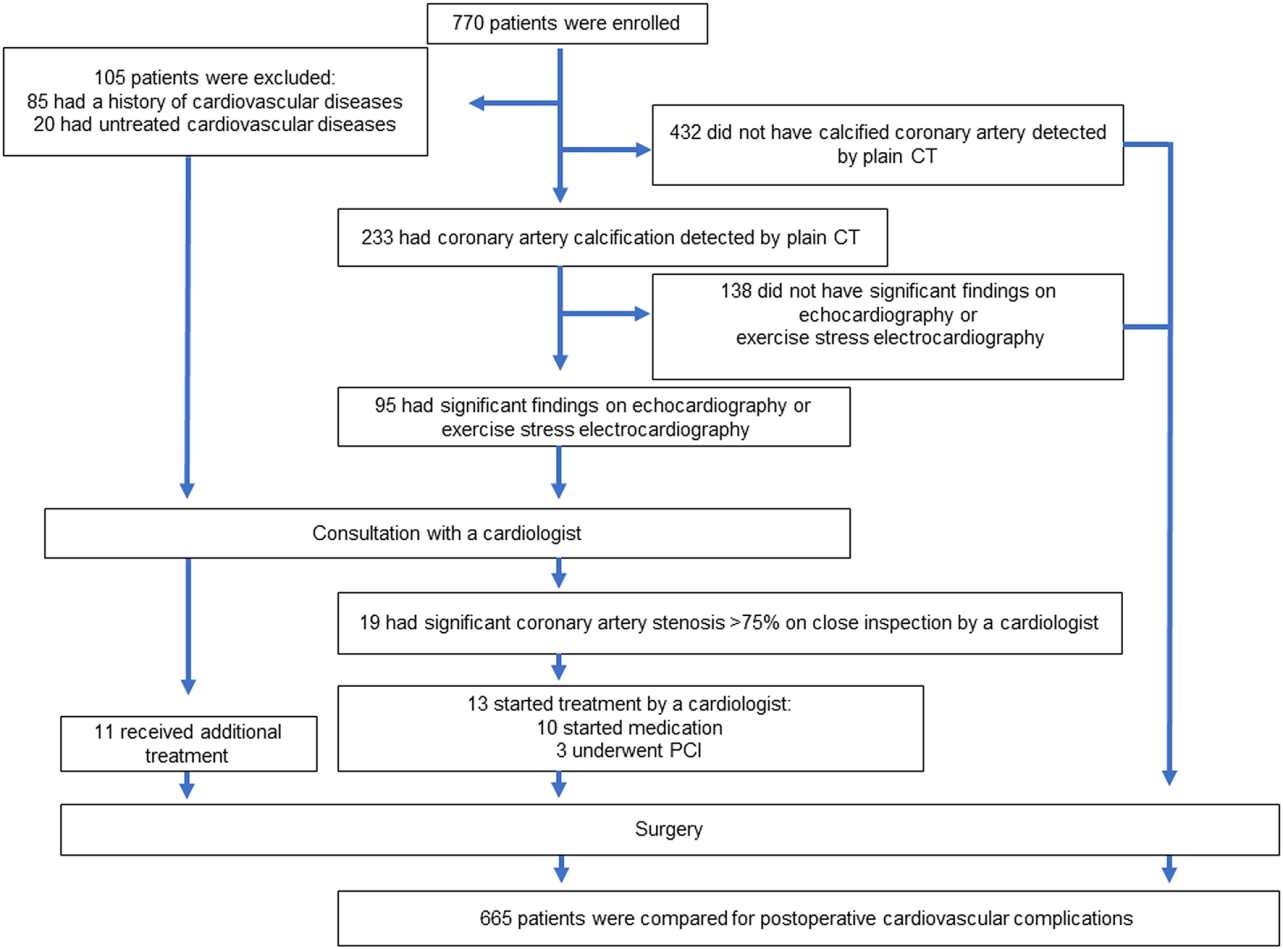
Study flowchart showing the clinical protocol followed in evaluating patients with coronary artery calcification detected on plain CT in our department. *CT* computed tomography; *PCI* percutaneous coronary angiography

Between April 2010 and March 2020, a total of 770 patients underwent anatomical pulmonary resection at our hospital. Extended surgeries, such as combined resection of adjacent organs, bi-lobectomy, and pneumonectomy, were not included in this study. Among the total patients, 105 who had been treated by a cardiologist or presented circulatory symptoms at the time of the first medical examination were introduced to a cardiologist before surgery as usual. The remaining 665 patients who had neither symptoms nor a history of cardiac disease without hypertension underwent resting electrocardiography before surgery. CAC was defined as high-concentration changes in the coronary arteries, ranging from spot-like to segmental changes, judged to be calcifications on plain chest CT with the mediastinal window. The presence or absence of calcification was determined by the attending physician. Patients in whom CAC was detected using plain chest CT underwent echocardiography and exercise stress electrocardiography along with resting electrocardiography. Among these patients, those who had significant echocardiography or exercise stress electrocardiography findings had consultations with a cardiologist and underwent close examinations. Patients who had significant findings on close inspections by a cardiologist started undergoing treatments preoperatively (Fig. 1).

### Analyses of separated patients

First, the data of patients who had already undergone cardiovascular intervention and those who had some cardiovascular symptoms at the first visit (105 patients) were analyzed separately.

### Analyses of patients with CAC detected using plain chest CT

Second, the participants, excluding those who had already undergone cardiovascular intervention and those who had some cardiovascular symptoms at the first visit (665 patients), were divided into 2 groups according to previously detected CAC using plain chest CT: the calcification (+) (233 patients) and calcification (−) (432 patients) groups. Subsequently, their age, sex, and medical history (hypertension, diabetes mellitus, hyperlipidemia, smoking, and antithrombotic therapy for ischemic diseases, such as cerebral infarction but excepting ischemic heart disease) were compared, and the presence of risk factors for CAC was examined.

### Echocardiography and exercise stress electrocardiography

Third, the 233 patients with CAC underwent echocardiography and exercise stress electrocardiography. Among them, 95 patients with significant findings were referred to a cardiologist and underwent close examinations, if required, as judged by the cardiologist. Among the 95 patients, 63 had abnormalities on echocardiography and exercise stress electrocardiography and 32 were unable to undergo exercise stress electrocardiography because of lower-limb paralysis caused by cerebrovascular disorders or lower-limb muscle weakness were considered to have significant findings and were referred to a cardiologist for consultation.

### Perioperative treatment and risk factors for perioperative cardiovascular complications

Finally, all patients (665 patients) with or without CAC were divided into 2 groups: those with cardiovascular complications during the perioperative period (21 patients) and those without such complications (644 patients). The age, sex, and medical history (hypertension, diabetes mellitus, hyperlipidemia, smoking, and antithrombotic therapy), as well as CAC detected on plain chest CT were compared between the two groups, and risk factors for perioperative cardiovascular complications were investigated. In addition, perioperative treatment for patients with significant coronary stenosis was investigated.

### Statistical analyses

Continuous variables are presented as the median value with 25th and 75th percentiles. Categorical data are presented as the percentage of patient numbers. We used the Mann–Whitney *U* test to compare the non-normally distributed continuous variables and the chi-square or Fisher’s exact test to compare the proportions of categorical variables between the two groups. To determine risk factors for CAC and cardiovascular accidents, a multivariate analysis was performed using multiple logistic regression. Continuous variables (age) were dichotomized with respect to the median value in the multivariate analysis. A *p* value < 0.05 was considered statistically significant. The JMP software program, version 15 (SAS Institute Inc., Cary, NC, USA), was used to perform all statistical analyses.

## Results

### Characteristics of the excluded patients

In total, 105 patients had been treated by a cardiologist previously (85 cases) or had circulatory symptoms at the time of the first medical examination (20 cases) (Table 1). Concerning the type of disease treated by the cardiologist (ischemic heart diseases, arrhythmia, and other various cases), 38 (44.7%) patients were treated for ischemic heart diseases. Among those who had a history or symptoms of heart disease, 79 (75.2%) patients had CAC. Of the patients with a history of ischemic heart disease, 34 (89.5%) had CAC. Among these patients, new or additional treatments were initiated preoperatively in 11 patients.

**Table 1:** Patients who consulted a cardiologist at the time of their first visit to our department

Under therapy (cases)	85	Untreated (cases)	20
Ischemic heart disease	38 (44.7%)	Symptoms	2
Post-coronary artery bypass grafting	5	Hypertension	6
Post-percutaneous coronary intervention	27	Resting ECG abnormalities	12
Others	6		
Arrhythmia	34 (40.0%)		
Atrial fibrillation	15		
Pacemaker indwelling	4		
Others	15		
Others	13 (15.3%)		
Valvular disease	2		
Cardiomyopathy	4		
Pulmonary embolism	1		
Pulmonary hypertension	1		
Cardiac sarcoidosis	1		
Aortic dissection	2		
Arteriosclerosis obliterans	2		

ECG, electrocardiography

In total, 105 patients had received treatment by a cardiologist (85 cases) or had circulatory symptoms at the time of the first medical examination (20 cases). Concerning the types of disease treated by a cardiologist (ischemic heart diseases, arrhythmia, and other various diseases), 38 patients were treated for ischemic heart diseases (44.7%)

### Characteristics of patients with CAC detected on plain chest CT

In this study, 432 patients did not have calcified coronary arteries on plain chest CT, and they underwent pulmonary resection with only electrocardiography at rest without close inspection before surgery. In contrast, 233 patients had calcified coronary arteries on plain chest CT, and they underwent echocardiography and exercise stress electrocardiography before surgery. The patient characteristics are presented in Table 2. The calcification (+) group had a higher number of older patients (≥ 75 years); male patients; and patients with hypertension, diabetes mellitus, anticoagulant use, and a history of smoking than the calcification (−) group in the univariate analysis (Table 3). In the multivariate analysis, study factors, except for sex, diabetes mellitus, hyperlypidemia, and smokers, were significantly higher in the calcification (+) than in the calcification (−) group (Table 3).

**Table 2:** Baseline characteristics of patients with or without coronary artery calcification among the 665 patients

Variables	Total (*n* = 665)	Calcification (+) (*n* = 233)	Calcification (−) (*n* = 432)	*p* value
Age (years), median (IQR)	69 (64–75)	74 (67–77)	67 (62–73)	< 0.001*
Sex Male, *n* (%)	405 (60.9%)	162 (69.5%)	243 (56.3%)	< 0.001*
Hypertension, *n* (%)	309 (47.2%)	132 (56.7%)	177 (41.0%)	< 0.001*
Diabetes mellitus, *n* (%)	127 (19.1%)	56 (24.0%)	71 (16.4%)	0.017*
Hyperlipidemia, *n* (%)	148 (22.3%)	61 (26.2%)	87 (20.1%)	0.74
Antithrombotic therapy, *n* (%)	54 (8.1%)	33 (14.2%)	21 (4.9%)	< 0.001*
Smoking, *n* (%)	405 (60.9%)	162 (69.5%)	243 (56.3%)	< 0.001*

IQR, interquartile range

*p < 0.05

**Table 3:** Factors associated with the risk of coronary artery calcification among the 665 patients according to the univariate and multivariate logistic regression analyses

Variables	Univariate analysis			Multivariate analysis		
	*b* (SE)	Crude OR (95% CI)	*p* value	*b* (SE)	Adjusted OR (95% CI)	*p* value
Age ≥ 75 years	1.39 (0.18)	4.04 (2.83–5.78)	< 0.001*	1.27 (0.19)	3.57 (2.46–5.17)	< 0.001*
Sex Male vs. female	0.57 (0.17)	1.77 (1.27–2.48)	< 0.001*	0.33 (0.21)	1.39 (0.93–2.09)	0.11
Hypertension	0.63 (0.16)	1.88 (1.36–2.60)	< 0.001*	0.39 (0.18)	1.47 (1.04–2.09)	0.03*
Diabetes mellitus	0.48 (0.20)	1.61 (1.09–2.38)	0.01*	0.36 (0.22)	1.44 (0.94–2.20)	0.097
Hyperlipidemia	0.34 (0.19)	1.41 (0.97–2.05)	0.074	0.31 (0.21)	1.36 (0.90–2.06)	0.14
Antithrombotic therapy	1.17 (0.30)	3.23 (1.82–5.73)	< 0.001*	0.87 (0.32)	2.40 (1.29–4.45)	0.006*
Smoker	0.57 (0.17)	1.78 (1.27–2.47)	< 0.001*	0.345 (0.21)	1.41 (0.94–2.12)	0.098

SE, standard error; CI, confidence interval; OR, odds ratio

The calcification (+) group had a higher predominance of older patients (≥ 75 years old); male patients; and patients with hypertension, diabetes mellitus, hyperlipidemia, a history of smoking, and anticoagulant use than the calcification (−) group. All study factors except for sex were significantly higher in the calcification (+) group than in the calcification (−) group

*p < 0.05

### Echocardiography and exercise stress electrocardiography

In this study, 138 of the 233 patients with CAC had no significant echocardiography or exercise stress electrocardiography findings, and they underwent pulmonary resection without close inspection before surgery. The remaining 95 patients, including 63 who had significant echocardiography findings (pericardial fluid, 2 patients; local hypokinesis, 9 patients) or exercise stress electrocardiography findings (pseudo-positive findings, 7 patients; ST change, 35 patients; ventricular premature contraction, 6 patients; abnormal Q wave, 2 patients; symptoms, 4 patients) (there was some overlap), or 32 patients with non-cardiac vascular disease who were unable to exercise as a result of lower-limb paralysis due to cerebrovascular disease or lower-limb muscle weakness associated with an impaired lower limb blood flow, thus suggesting the presence of asymptomatic ischemic heart disease. These patients were referred to a cardiologist who performed a closer examination, if required, before surgery.

### Treatment by a cardiologist in the perioperative period

In total, 95 patients were referred to a cardiologist. Of them, 29 did not require close inspection, 7 underwent myocardial scintigraphy, 48 underwent coronary CT angiography, and 22 underwent CAG (there was some overlap). Nineteen (20.0%) of the 95 patients showed significant stenosis of the coronary arteries (≥ 75%), accounting for 8.2% of all 233 patients with CAC. The sites of CAC and the actual coronary artery stenosis were the same in 14 patients; however, in a few patients, stenosis was observed in sites without calcification (patients 1 and 2 had complete mismatch; patients 9, 15, and 17 had some mismatch; Table 4).

**Table 4:** Findings for individual patients who consulted a cardiologist during the perioperative period

Patient no	Pathology	c-Stage	Operative method	Calcification site	Calcification range	Coronary stenosis	Preoperative treatment	Preoperative intervention period for cardiology (days)	Intraoperative treatment	Postoperative treatment	Period from surgery to PCI (days)
1	Ad	IB	LUL	LAD	Segmental	RCA #2 75%	Medication	14	–	#2 BMS	64
2	Ad	IA2	LUL	LMT	Segmental	RCA #2 75%	#2 DES	56	–	Medication	–
3	Sq	IA3	RUL	RCA, LAD	Segmental	RCA #4PD 75%, LAD #7 90%	#7 DES #9 POBA	51	–	#7, #9 POBA	113
4	Ad	IA2	RUL	RCA, LAD	Segmental	LAD #6 75%	Medication	34	–	#6 POBA, DES #9 POBA, 4AV POBA, DES	87
5	Ad	IB	LUL	LAD	Segmental	LAD #7 50–75%	Medication	27	–	#7 DES	61
6	Ad	IIB	LUL PA plasty	LAD	Segmental	LAD #7 75%	Medication	30	–	Medication	–
7	Ad	IIB	RUL	RCA, LAD, Cx	Segmental	RCA #2 75% #3 100% LAD #6 75% #7 50%	#2 POBA	38	Vasodilator	#2 DES #3 DES #6 DES #7 DES	235
8	Ad	IIA	RUL	RCA, LAD	Segmental	RCA #1 90% LAD #7 90% #10 75%	Medication	7	Vasodilator	#1 planned PCI #7 DES #10 POBA	35
9	Ad	IA2	RLL	LAD, Cx	Segmental	RCA #2 75% Cx #14 90%	Medication	54	Vasodilator	Planned PCI	–
10	Ad	IB	RLL	RCA, LAD	Segmental	RCA #2 50–75% LAD #7.9 75%	Medication	20	–	#7 DES #2 DES	59
11	Ad	IA3	RUL	RCA, LAD, Cx,	Segmental	RCA #3 75%	-	31	–	#3 DES	162
12	Sq	IIIA	RUL	LAD	Segmental	LAD 75%	Medication	12	–	#2 DES	57
13	Pulmonary sequestration	RLL	LAD	Segmental	LAD 50–75%	Medication	40	-	CAG follow-up	–	
14	Ad	IIB	LUL	LAD	Segmental	LAD #6.7 90%	Medication	10	Vasodilator	#6, 7 DES	38
15	Sq	IA2	LUL	RCA, LAD, Cx,	Segmental	RCA 50% Cx HL 75%	-	28	Vasodilator	–	–
16	Ad	IIB	LLL	RCA, LAD, Cx,	Segmental	RCA #1 50%, #2 75%	-	28	–	Medication	–
17	Sq	IA3	LUL	RCA, LAD, Cx,	Segmental	RCA #4PD 75%, LAD #9 50% Cx 25%	–	6	–	–	–
18	Ad	IIIA	RLL	RCA, LAD, Cx,	Segmental	LAD #10 75% Cx #14 75%	–	8	–	–	–
19	Sq	IA3	LLL	LAD	Segmental	LAD #6 50% #9 75%	–	4	–	–	–

Ad, adenocarcinoma; 4AV, atrioventricular branch; BMS, bare metal stent; CAG, coronary angiography; Cx, left circumflex coronary artery; DES, drug-eluting stent; HL, high lateral branch; LAD, left anterior descending coronary artery; LLL, left lower lobectomy; LUL, left upper lobectomy; LMT, left main trunk; PA, pulmonary artery; PCI, percutaneous coronary intervention; 4PD, posterior descending branch; POBA, plain old balloon angioplasty; RCA, right coronary artery; RLL, right lower lobectomy; RUL, right upper lobectomy; Sq, squamous cell carcinoma

Nineteen patients had significant preoperative coronary artery stenosis, of whom 13 received preoperative therapeutic intervention, and 4 received intraoperative vasodilators to prevent coronary stenosis

In total, 13 of 19 patients (68.4%) with significant stenosis of the coronary arteries underwent preoperative treatment by cardiologists; 10 patients started taking medications, whereas 3 underwent percutaneous coronary intervention (PCI). One patient underwent plain old balloon angioplasty (POBA), one received drug-eluting stents (DESs), and one received both POBA and DESs. In addition, among those without significant coronary artery stenosis, six patients initiated new drug therapy, according to their symptoms.

In the intraoperative period, four patients received vasodilators to avoid coronary occlusions. The median time from cardiovascular intervention to surgery was 27.5 (4–56) days, and the patients with DESs underwent surgery 51 and 56 days later with simultaneous anticoagulant therapy in the perioperative period. In the postoperative period, observation was continued in 5 patients (1 patient was followed up with CAG), 3 patients continued taking medication only, and 10 underwent PCI (Table 4).

### Perioperative cardiovascular complications

Among the 665 patients with or without CAC, 1 patient (0.15%) had Grade IIIa cardiovascular complications based on the Clavien–Dindo classification (Grade IIIa atrial fibrillation [AF] requiring ablation). Twenty patients (3.0%) had mild cardiovascular complications below Grade II based on the Clavien–Dindo classification (Table 5). Overall, 57.1% of patients who developed perioperative complications had CAC, whereas 66.7% of patients who developed AF had CAC (Table 5). Patients with CAC were significantly more likely to develop postoperative AF than those without CAC (*p* = 0.02). No coronary accidents were recorded, and no perioperative cardiovascular complications were observed in the 25 patients in whom preoperative therapeutic intervention was performed or coronary artery stenosis was identified before surgery.

**Table 5:** Complications among the 665 patients

Description	Clavien–Dindo classification Grade	Number of patients	Number of patients with CAC
AF	I	2	1 (50%)
	II	12	9 (75%)
	IIIa	1	0 (0%)
PSVT	II	2	0 (0%)
Sinus tachycardia	I	2	1 (50%)
	II	1	0 (0%)
Bradycardia	II	1	1 (100%)
Total		21	12 (57.1%)

AF, atrial fibrillation; CAC, coronary artery calcification; PSVT, paroxysmal supraventricular tachycardia

The 665 patients were divided into the cardiovascular complication (+) group (21 patients) and the cardiovascular complication (−) group (644 patients). The patient characteristics according to the two groups are presented in Table 6. In the univariate analysis, the cardiovascular complication (+) group had a higher proportion of calcified coronary arteries on plain CT than those in the cardiovascular complication (−) group (Table 7). In the multivariate analysis, the proportion of patients who showed a higher tendency to have calcified coronary arteries detected on plain CT was significantly higher in the cardiovascular complication (+) group than in the cardiovascular complication (−) group (Table 7).

**Table 6:** Baseline characteristics of patients with or without perioperative cardiovascular complications among the 665 total patients

Variables	Total (*n* = 665)	Perioperative cardiovascular complication (+) (*n* = 21)	Perioperative cardiovascular complication (−) (*n* = 644)	*p* value
Age (years), median (IQR)	69 (64–75)	68 (64.75–75.25)	69 (63.5–75)	0.60
Sex Male, *n* (%)	405 (61.8%)	15 (71.4%)	390 (60.6%)	0.32
Hypertension, *n* (%)	309 (47.2%)	12 (57.1%)	297 (46.1%)	0.32
Diabetes mellitus, *n* (%)	127 (19.1%)	4 (19.0%)	123 (19.1%)	0.99
Hyperlipidemia, *n* (%)	148 (22.3%)	7 (33.3%)	141 (21.9%)	0.21
Antithrombotic therapy, *n* (%)	54 (8.2%)	3 (14.3%)	51 (7.9%)	0.29
Smoker, *n* (%)	396 (59.5%)	15 (71.4%)	381 (59.2%)	0.26
Coronary calcification, *n* (%)	233 (35.6%)	13 (61.9%)	220 (34.2%)	0.0087*

IQR, interquartile range

*p < 0.05

**Table 7:** Factors associated with a risk of perioperative cardiovascular complications among the 665 patients according to univariate and multivariate logistic regression analyses

Variables	Univariate analysis			Multivariate analysis		
	*b* (SE)	Crude OR (95% CI)	*p* value	*b* (SE)	Adjusted OR (95% CI)	*p* value
Age ≥ 75 years	0.29 (0.47)	1.34 (0.53–3.38)	0.53	0.089 (0.50)	1.09 (0.41–2.92)	0.86
Sex Male vs. female	0.49 (0.49)	1.63 (0.62–4.25)	0.32	− 0.06 (0.72)	0.94 (0.22–3.86)	0.93
Hypertension	0.44 (0.45)	1.56 (0.65–3.75)	0.32	− 0.21 (0.46)	0.81 (0.32–2.01)	0.65
Diabetes mellitus	− 0.0034 (0.56)	0.99 (0.33–3.01)	0.99	0.23 (0.58)	1.26 (0.40–3.96)	0.69
Hyperlipidemia	0.58 (0.47)	1.78 (0.71–4.50)	0.22	− 0.51 (0.49)	0.59 (0.23–1.57)	0.29
Antithrombotic therapy	0.66 (0.64)	1.94 (0.55–6.79)	0.30	− 0.36 (0.66)	0.69 (0.19–2.58)	0.59
Smoker	0.55 (0.48)	1.73 (0.66–4.51)	0.27	− 0.42 (0.73)	0.65 (0.16–2.73)	0.57
Coronary calcification	1.14 (0.46)	3.13 (1.28–7.67)	0.01*	− 1.02 (0.49)	0.36 (0.14–0.94)	0.038*

SE, standard error; CI, confidence interval; OR, odds ratio

Coronary calcification was a significant risk factor in the univariate and multivariate analyses

*p < 0.05

## Discussion

In this study, we revealed that patients with CAC detected on plain chest CT have many risk factors for cardiovascular disease and have a high risk of perioperative cardiac complications despite having no symptoms in the preoperative period. However, asymptomatic patients with significant stenosis of the coronary arteries who underwent appropriate preoperative intervention had no perioperative complications. Selective scrutiny of patients with CAC using preoperative plain chest CT and appropriate preoperative intervention may reduce the risk of perioperative cardiac complications.

The United States (US) Preventive Task Force suggests that the current evidence regarding CAC is insufficient to weigh the benefits and risks of adding it to traditional risk assessment measures for heart attack and stroke prevention in people without symptoms because of the lack of access to original data [6]. In addition, a review of CAC in lung cancer screening suggests that CAC is a less accurate test for cardiovascular risk than extra-coronary thoracic arterial calcifications (ECC) and that the measurement of ECC may be a better indicator of cardiovascular risk [7]. However, compared with ECC, CAC is more strongly associated with coronary heart disease [7], and the presence of CAC on plain chest CT may be a predictor of a high risk of chronic coronary artery stenosis [8]. Our study also showed that patients with a history of ischemic heart disease had a higher rate of CAC than those without such a history.

In addition, CAC is independently associated with an increased risk of AF [9], and even patients without coronary artery disease (CAD) have an increased risk of stroke and death if they have AF and incidental CAC detected on chest CT [10]. Although arrhythmias, including AF, are common complications after pulmonary resection, it is important to know preoperatively which asymptomatic patients are at an increased risk of developing a cardiac disease. According to our study, patients with asymptomatic CAC had a significantly higher tendency to have many of the risk factors for coronary artery stenosis presented by the US Preventive Services Task Force [6] than those without asymptomatic CAC. In addition, only CAC was identified as a risk factor for perioperative complications, while among the patients who developed perioperative complications, those with AF were likely to have CAC. Even when the risk factors appear to be controlled, each risk accumulated over time may be reflected in CAC. Thus, CAC may be the most reliable factor to consider when evaluating patients referred for surgical treatment at tertiary medical institutions.

In addition, in this study, CAC was not scored, but all cases of CAC, from spot calcifications to segmental calcifications, were included. Although all cases with significant coronary artery stenosis detected at CAG had segmental CAC in this study, in some cases, the site of CAC did not match the actual site of coronary artery stenosis. In studies using coronary arteriography, intracoronary ultrasonography, and autopsy findings, the CAC scores were correlated well with atherosclerotic plaque burden; however, there was no association with the luminal cross-sectional area. The presence of calcification detected by chest CT does not imply significant coronary stenoses [11]. However, to identify cases with a high risk of cardiovascular complications, scrutinizing patients with CAC detected on plain chest CT is important.

The sensitivity and specificity of exercise stress electrocardiography when searching for coronary artery stenosis are approximately 70% and 75%, respectively [12–14]. In addition, in the present study, patients with lower-limb movement disorders due to medical issues, such as arthropathy, arteriosclerosis obliterans, and cerebrovascular disorders, who did not undergo exercise stress electrocardiography were considered as high-risk patients and selected for a close examination by cardiologists. In fact, 5 of the 32 patients who could not undergo stress electrocardiography had significant stenosis in the coronary arteries. In contrast, resting echocardiography is useful for patients with chronic CAD, especially for those with hypertension or valvular disease [15, 16]. Cardiac expansion or a decreased cardiac function detected on resting echocardiography in cases of chronic CAD is associated with the prognosis [17, 18].

Patients with CAD in pulmonary surgery are more likely to have perioperative complications, including coronary vascular events than patients without CAD, although this does not affect long-term outcomes after pulmonary resection [19]. Furthermore, in the LIFE-Heart Study, coronary atherosclerosis (CAS) without CAD (stenosis of the coronary arteries < 75%) was associated with an increased risk of AF [20]. In the present study, perioperative complications did not occur in patients with CAC in whom preoperative scrutiny was performed and led to appropriate therapeutic intervention. Identification and therapeutic intervention in patients with CAC who had asymptomatic CAD or CAS may have reduced perioperative complications.

Rather than scoring CAC, screening patients with CAC by echocardiography and exercise stress electrocardiography and using the results to perform a cardiological examination even if the patient is asymptomatic would allow us to intervene preoperatively in patients with cardiovascular risk, even without access to special equipment to quantify and score CAC. This could potentially reduce perioperative complications.

In patients with a DES, dual antiplatelet therapy for more than one year is recommended [21, 22], and its discontinuation for non-cardiac surgery prior to this time point is associated with an increased risk of major adverse cardiac events [23, 24]. However, surgery in the presence of dual antiplatelet therapy may increase the risk of perioperative bleeding and its associated complications [25, 26]. Therefore, surgery for lung disease must be delayed for an extended period. In our department, after consultation with a cardiologist, balloon dilatation and drug treatment are generally initiated before surgery with consideration of the degree of pulmonary lesion progression, degree of stenosis of the coronary artery, and site of stenosis; preoperative coronary stenting is performed only in patients with severe coronary artery stenosis. Patients who underwent preoperative stenting received surgery while maintaining anticoagulation in the perioperative period. High-risk patients who did not undergo preoperative stenting received surgery under intraoperative coronary vasodilation after consultation with the anesthesiologist. The DES was then implanted when patients had stabilized after surgery. For the maintenance of anesthesia, it was useful for the anesthesiologist to be aware of the presence of coronary stenosis. Perioperative coronary complications did not occur in this study. By preoperatively identifying patients with significant coronary artery stenosis, it may be possible to prevent cardiovascular complications through appropriate preoperative and perioperative management, including intraoperative anesthesia management, even in patients with risk factors for cardiovascular complications.

### Limitations

Several limitations associated with the present study warrant mention. In this study, no instances of serious perioperative coronary complications were observed. However, we did not perform perioperative troponin *T* measurement or postoperative electrocardiography retesting, which might have concealed potential coronary complications in the perioperative period. It is also possible that patients with potential asymptomatic CAD were present in the CAC (−) group, as a close examination was not performed in this group. In addition, our study’s results deviated from those previously proposed by the US Preventive Task Force, where the current evidence regarding CAC was insufficient to weigh the benefits and risks of adding it to traditional risk assessment measures for heart attack and stroke prevention in asymptomatic patients. Considering the difference in findings between the present and previous studies, it is reasonable to assume that this cohort exhibits a selection bias, possibly because of the involvement of tertiary medical institutions, surgical referrals, and the single-center setting. Patients who might have been deemed unfit for surgery because of the presence of risk factors, such as elderly persons, hypertension, diabetes, antithrombotic therapy, or smoker, might have already been excluded. However, CAC was confirmed as a clear risk factor in the present study. Future studies should include patients with CAC without scrutiny or those without CAC with scrutiny who underwent lung surgery. However, based on the present findings, we believe that not performing a thorough examination on patients with CAC may be ethically problematic and requires careful consideration.

In conclusion, patients with CAC are more likely to have cardiac risk factors than those without CAC. CAC is a risk factor for perioperative cardiac complications, and the selective evaluation of and intervention for patients with CAC using preoperative chest CT may reduce the risk of perioperative cardiac complications.
